# A phase I single-blind clinical trial to evaluate the safety of oil palm phenolics (OPP) supplementation in healthy volunteers

**DOI:** 10.1038/s41598-018-26384-7

**Published:** 2018-05-29

**Authors:** Syed Fairus, Soon-Sen Leow, Isa Naina Mohamed, Yew-Ai Tan, Kalyana Sundram, Ravigadevi Sambanthamurthi

**Affiliations:** 1 Malaysian Palm Oil Board (MPOB), No. 6 Persiaran Institusi, Bandar Baru Bangi, 43000 Kajang, Selangor Malaysia; 2 Universiti Kebangsaan Malaysia Medical Centre (UKMMC), Jalan Yaacob Latif, Bandar Tun Razak, 56000 Cheras, Kuala Lumpur Malaysia; 3 Malaysian Palm Oil Council (MPOC), 2nd Floor, Wisma Sawit, Lot 6, SS6, Jalan Perbandaran, 47301 Kelana Jaya, Selangor Malaysia

## Abstract

Plant phenolics are being increasingly consumed globally with limited scientific and clinical evidence pertaining to safety and efficacy. The oil palm fruit contains a cocktail of phenolics, and palm oil production results in high volumes of aqueous by-products enriched in phenolics and bioactives. Several lines of evidence from *in vitro* and *in vivo* animal studies confirmed that the aqueous extract enriched in phenolics and other bioactives collectively known as oil palm phenolics (OPP) is safe and has potent bioactivity. A phase one clinical trial was conducted to evaluate the safety and effects of OPP in healthy volunteers. In this single-blind trial, 25 healthy human volunteers were supplemented with 450 mg gallic acid equivalent (GAE)/day of OPP or control treatments for a 60-day period. Fasting blood and urine samples were collected at days 1, 30 and 60. Medical examination was performed during these trial interventions. All clinical biochemistry profiles observed throughout the control and OPP treatment period were in the normal range with no major adverse effect (AE) or serious adverse effect (SAE) observed. Additionally, OPP supplementation resulted in improvement of total cholesterol and LDL-C levels, compared to the control treatment. The outcomes support our previous observations that OPP is safe and may have a protective role in reducing cholesterol levels.

## Introduction

Phenolics are believed to be major contributors to the disease-protective effects of fruit and vegetables. They are important naturally occurring antioxidants and broadly characterized as aromatic metabolites that possess one or more ‘acidic’ phenolic hydroxyl groups^1^. Phenolics are mainly categorized into two classes; namely flavonoids and phenolic acids. Several types of flavonoids have been identified comprising flavones, flavonols, flavanols, flavanones, isoflavones, proanthocyanidins and anthocyanins. Several phenolic acid compounds such as caffeic, chlorogenic and ferulic acids are commonly found in daily food and beverages.

The oil palm fruit contains numerous phenolic compounds. During the extraction of palm oil from the oil palm fruit bunch, a large volume of water-soluble by-products rich in phenolic compounds is generated and discarded in the aqueous waste stream. Several phenolic compounds have been identified in the aqueous phase such as protocatechuic acid, *p*-hydroxybenzoic acid and three isomers of caffeoyl-shikimic acid^2^. Previously we successfully developed and patented a novel process to recover an extract enriched in phenolics and other bioactive compounds from the aqueous by-product^3–5^.

Condensed from the literature, a number of physiological and therapeutic effects of phenolics have been demonstrated. Phenolics may reduce cholesterol absorption due to the interaction of these compounds with cholesterol carriers and transporters present across the brush border membrane^6^. Several *in vitro* studies suggested that phenolics inhibit LDL-oxidation^7,8^ and aggregation of platelets^9^. OPP exhibits free-radical scavenging activity, by acting as a hydrogen donor and has been demonstrated to scavenge DPPH (2,2-diphenyl-picryl-hydrazyl) free radicals^2,10^. In mouse cell culture studies, OPP inhibited the progression of various cancer cell lines^2^. Potent anti-cancer activity of OPP was indicated in BALB/c mice injected with myeloma cells. The tumor numbers in the mice supplemented with OPP as a drink were significantly lower compared to controls^11^. Microarray profiling of BALB/c mice supplemented with OPP showed that several genes related to hepatic lipid catabolism were up-regulated, indicating suppression of liver fat and visceral fat accumulation in the body. Additionally, genes involved in cholesterol biosynthesis were down-regulated, suggesting a possible role in preventing hypercholesterolemia^12^. OPP also attenuated atherosclerosis^13,14^ as well as responded positively towards the distal colonic contractility and motility^15^ in animal models.

Long-term intake of OPP protected healthy, young Nile rats against diabetes onset, as measured by glucose, blood lipids, and weights of livers and kidneys^11^. These outcomes were probably due partly at least to the phenolic compounds in OPP and their protection of ß-cells in the pancreatic islets against oxidative stress, thereby maintaining the integrity and ability of ß-cells to produce insulin^16^. Recently, the anti-diabetic potential by OPP supplementation has been postulated to be a result of enhanced insulin sensitivity and reduced glucose absorption/output and not an increase in insulin secretion, as indicated by down-regulation of insulin signaling genes^17^.

The wide spectrum of bioactivities demonstrated by OPP in *in vitro* and *in vivo* systems suggests its potential application for a range of chronic diseases. Currently however, there is still no clinical data on the effects of OPP in humans in terms of safety and efficacy. Therefore, we conducted a phase one clinical trial to evaluate the physiological effects of OPP in healthy human subjects. The primary objective of this trial was to evaluate if there were any Adverse Effects (AE), and/or Serious Adverse Effects (SAE) following OPP administration (up to 60 days supplementation). Secondary objectives were to evaluate physiological effects which resulted from OPP supplementation. Data from this trial would be important for regulatory requirements and developing a safety profile of OPP besides understanding the physiological roles of OPP in humans under normal conditions.

## Methods

### Volunteers

Twenty-five healthy volunteers, consisting of 11 males and 14 females were recruited from the Malaysian Palm Oil Board (MPOB). Volunteers were thoroughly briefed on the objectives, design, and trial protocol before they signed the informed consent forms. All volunteers were normolipaemic, nonsmokers, and did not show any clinical symptoms associated with cardiovascular disease. They were examined for their health status and medical history by a medical officer before participation. Through the administration of a questionnaire and dietary interview, we established that none of the volunteers consumed any vitamin or herbal supplements, or were taking any prescribed medication. Female volunteers were not pregnant, lactating, or taking contraceptives at the time of recruitment. The recruitment was completed with the following baseline characteristics: Means ± SDs: Age, 29.24 ± 4.31 y; body mass index, 22.41 ± 3.99 kg/m^2^ (Table 1). The trial was approved by the Medical Research Ethics Committee (MREC), Malaysian Ministry of Health (NMRR-08-1616-3108) and registered with the Australian New Zealand Clinical Trial Registry (ANZCTR) database: www.anzctr.org.au/ (Trial Reference No: ACTRN 12611001122943, registration date: 27 Oct 2011). This registry is recognised by the World Health Organization International Clinical Trial Registry Platform (WHO ICTRP) as a Primary Registry. All methods and protocols were performed in accordance with the Declaration of Helsinki, The International Conference of Harmonisation (ICH) of Technical Requirements for Human Use and Good Clinical Practice (GCP).

**Table 1:** Volunteers demographic characteristics. Data is tabulated as mean ± SD, or n (%).

	OPP treatment (n = 25)	Control treatment (n = 25)
Age (years)	29.24 ± 4.31	
Sex		
Male	18 (72%)	
Female	17 (68%)	
Body mass index (kg/m^2^)	22.41 ± 3.99	

### Study protocol

The trial was a single-blind trial, with comparison to control treatment. The study design enabled each subject to serve as his/her own control. On day one of the trial, volunteers attended a clinic for baseline measurement of plasma clinical profiles, after an overnight fast of at least 10 hours. Fasting blood (20 mL) and urine samples were taken. Volunteers were assigned into two intervention groups, where one group (treatment group) was given 150 mL of OPP (containing 1500 mg/L GAE OPP), twice per day. The total amount of phenolic compounds supplemented was 450 mg GAE/day. The composition of OPP was analyzed according to the method described previously^2^. Concentrations of major phenolic compounds are tabulated in Table 2. The other group (control group), was given 150 mL of control drinks (drinking water), twice per day. Both treatment and control drinks were made available in 250 mL amber glass bottles and kept refrigerated until distribution to the volunteers. In order to ensure compliance, all volunteers consumed both drinks in front of the investigator. All drinks consumption record was verified by the investigator and documented accordingly. The trial design allowed the investigators to detect early physiological outcomes from OPP intake. The trial period for OPP and control treatments was 60 days each where a four-week (28 days) wash-out period was allowed between both treatments. Volunteers were allowed to maintain their habitual diets and life styles during this period. Fasting blood (20 mL) and urine were also sampled at day 30 and 60. In every clinic visit, volunteers were thoroughly examined for their health status by the medical officer. The measurement of body weight (Table 3) and blood pressure (Table 4) was documented before each bleeding session.

**Table 2:** Mean concentrations of major phenolic compounds in oil palm phenolics (OPP).

	Concentration (mg/L)
Protocatechuic acid	50
*p*-Hydroxybenzoic acid	577
Caffeoylshikimic acid	890
Total major phenolics	1517
Gallic acid equivalent (GAE)	1500
Amount supplemented to volunteers per day (150 mL OPP, twice per day)	450 mg/day

**Table 3:** Body weight changes in volunteers (mean ± SD).

	OPP treatment (n = 25)	Control treatment (n = 25)
Body weight (kg)		
Day 1	60.38 ± 14.2	60.4 ± 13.52
Day 30	60.35 ± 14.14	60.21 ± 13.79
Day 60	60.41 ± 14.0	60.2 ± 13.69

**Table 4:** Blood pressure profiles (mean ± SD).

	OPP treatment (n=25)	Control treatment (n=25)
Systolic (mm Hg)		
Day 1	117.60 ± 13.32	115.20 ± 10.46
Day 30	112.24 ± 11.35	112.64 ± 12.08
Day 60	114.64 ± 10.32	112.64 ± 13.26
Diastolic (mm Hg)		
Day 1*	79.92 ± 7.91	76.40 ± 7.44
Day 30	74.48 ±7.33	74.24 ± 7.38
Day 60	74.32 ± 7.02	76.88 ± 8.81
Pulse rate (bpm)		
Day 1	68.72 ± 3.26	69.12 ± 3.96
Day 30	70.56 ± 3.81	71.04 ± 4.62
Day 60	68.80 ± 4.04	69.04 ± 3.61

*Baseline diastolic level (day 1) was significantly different between treatments, p = 0.043 (Wilcoxon’s signed-rank test). Values were further used as covariate in repeated measures. The time × treatment analysis was however not significant, p = 0.118 (2-factor repeated measures ANOVA).

Blood samples were drawn from the volunteers by an experienced and well-trained phlebotomist using syringes. A 20 mL blood sample was then transferred into blood collection tubes either with or without ethylenediamine tetra acetic acid (EDTA). The EDTA treated blood samples were centrifuged at 3000 × g for 20 minutes at 7° C to obtain plasma samples. Urine samples were also taken prior to the blood sampling. Samples were immediately analyzed by Cobas 8000- Integrated chemistry and immunoassay Electrochemiluminescence–ECLIA platform (Roche, Indianapolis USA), Immulite 2000 XPi-Chemiluminescence immunoassay platform (Siemens, Erlargen, Germany), Urisys 2400-Fully automated Urine FEME analyzer (Roche, Indianapolis, USA) and Cell-Dyn Ruby–Fully automated 5-part hematology analyser (Abbott, Illinois, USA) for clinical biochemistry profiles (as tabulated in Table 5 to Table 11). A comprehensive clinical trial protocol of the study is provided in the Supplementary File.

### Statistical analysis

Wilcoxon-Signed Test was performed to compare significance of differences between parameters of interest before (baseline, day 1) and after treatment (days 30, and 60) for each treatment. Effects of treatment on parameters of interest were analyzed for their time × treatment interaction, using Two-factor repeated measures analysis of variance (ANOVA) with an interaction term to detect whether there was a significant difference of plasma and urine profiles between OPP and control treatments. If there was any significant time × treatment interaction, the value at the specific day of treatment was extensively compared using Wilcoxon-Signed Rank Test. To increase the stringency of the analysis, Bonferroni correction for multiple testing was applied. Statistical analysis was performed using Statistical Package for Social Sciences (SPSS®) for WINDOWS software (Version 10.0, SPSS Inc. Chicago, USA) and MS Excel 2003 (Microsoft Corp. California, USA). The MS Excel software was used for tabulation of statistical charts. The SPSS® software was utilized for calculation of plasma profiles and analyses of Repeated Measures ANOVA, Bonferroni and Wilcoxon-Signed Rank Test. Values were considered significant at *P* < 0.05.

## Results

All 25 volunteers completed the trial. Table 1 shows the demographic characteristics of the volunteers. The trial was completed with the following baseline characteristics: Means ± SDs: Age, 29.24 ± 4.31 y; body mass index, 22.41 ± 3.99 kg/m^2^. There was no change in body weight of all volunteers (Table 3). During the trial, there was no dropout and none of the volunteers demonstrated any adverse effects (AE) or serious adverse effects (SAE).

Sample size or number of volunteers was determined based on calculation of Effect Size (ES). Data from other human studies investigating the antioxidant effects of phenolic compounds on several plasma biochemistry profiles^18–21^ were pooled and calculated for their ES. Estimation of sample size was conducted by introducing the ES value into the Altman Normogram. Calculations and statistical analysis for this part were carried out according to previous statistical methods^22,23^. This analysis indicated that the minimum number of volunteers required for this study was 23 per group. Hypothetically, this number would allow investigators to observe any difference between control and treatment groups.

All clinical biochemistry parameters including blood pressure (Table 4), liver function (Table 5), renal function (Table 6), haematology (Table 7), lipid profiles (Table 8), glucose and insulin levels (Table 9), hsCRP, whole blood HbA1C, urine albumin, ACR profile (Table 10) and electrolytes (Table 11) were in the normal range according to standard clinical safety references. There were no significant differences in any of these clinical biochemistry profiles between OPP and control treatments during the whole intervention period.

**Table 5:** Plasma liver function profiles (mean ± SD).

	OPP treatment (n = 25)	Control treatment (n = 25)
Total protein (g/L)		
Day 1	77.72 ± 3.85	77.88 ± 3.77
Day 30	79.28 ± 3.37	78.04 ± 4.99
Day 60	78.80 ± 4.75	79.84 ± 3.87
Albumin (g/L)		
Day 1	45.12 ± 1.81	45.08 ± 3.30
Day 30	46.36 ± 1.87	45.72 ± 2.46
Day 60	45.84 ± 2.69	46.24 ± 2.07
Globulin (g/L)		
Day 1	32.60 ± 4.12	32.80 ± 3.25
Day 30	32.92 ± 3.58	32.28 ± 4.01
Day 60	32.96 ± 3.98	33.60 ± 3.61
ALP (U/L)		
Day 1	62.32 ± 18.65	65.16 ± 15.79
Day 30	69.40 ± 19.93	68.28 ± 16.05
Day 60	63.64 ± 17.22	64.00 ± 16.30
AST (U/L)		
Day 1	19.92 ± 4.31	19.68 ± 5.43
Day 30	21.00 ± 3.50	21.04 ± 3.61
Day 60	20.00 ± 6.76	18.92 ± 3.76
ALT (U/L)		
Day 1	19.92 ± 11.24	18.76 ± 11.71
Day 30	19.60 ± 9.53	18.96 ± 7.35
Day 60	20.12 ± 13.48	18.16 ± 9.09
GGT (U/L)		
Day 1	21.04 ± 13.16	20.40 ± 11.38
Day 30	23.60 ± 11.92	22.68 ± 14.47
Day 60	22.48 ± 12.57	23.16 ± 13.60
Total bilirubin (µmol/L)		
Day 1	10.28 ± 4.14	9.28 ± 3.60
Day 30	10.28 ± 4.42	12.20 ± 5.91
Day 60	11.56 ± 5.49	12.36 ± 5.99

**Table 6:** Plasma renal function profiles (mean ± SD).

	OPP treatment (n = 25)	Control treatment (n = 25)
Urea (mmol/L)		
Day 1	3.87 ± 0.98	3.99 ± 0.92
Day 30	4.10 ± 1.11	3.81 ± 0.84
Day 60	4.07 ± 0.89	3.85 ± 0.91
Creatinine (µmol/L)		
Day 1	68.72 ± 15.27	68.96 ± 15.16
Day 30	72.72 ± 16.64	70.08 ± 15.62
Day 60	72.84 ± 15.77	71.00 ± 17.07
Uric acid (g/L)		
Day 1	0.33 ± 0.09	0.31 ± 0.08
Day 30	0.33 ± 0.10	0.32 ± 0.08
Day 60	0.33 ± 0.09	0.33 ± 0.10
Corrected calcium (mmol/L)		
Day 1	2.21 ± 0.06	2.21 ± 0.07
Day 30	2.20 ± 0.06	2.18 ± 0.07
Day 60	2.20 ± 0.09	2.21 ± 0.10
Phosphate (mmol/L)		
Day 1	1.10 ± 0.08	1.10 ± 0.13
Day 30	1.12 ± 0.14	1.11 ± 0.14
Day 60	1.14 ± 0.13	1.17 ± 0.11

**Table 7:** Haematology profiles (mean ± SD).

	OPP treatment (n = 25)	Control treatment (n = 25)
Haemoglobin (g/L)		
Day 1	137.36 ± 20.47	137.48 ± 20.26
Day 30	141.04 ± 20.25	137.64 ± 19.98
Day 60	138.56 ± 18.51	137.44 ± 19.94
RBC (×10^12^/L)		
Day 1	5.08 ± 0.48	5.04 ± 0.47
Day 30	5.13 ± 0.48	5.02 ± 0.46
Day 60	5.01 ± 0.42	5.05 ± 0.47
PCV (L/L)		
Day 1	0.43 ± 0.05	0.42 ± 0.05
Day 30	0.43 ± 0.05	0.42 ± 0.05
Day 60	0.43 ± 0.05	0.43 ± 0.05
MCV (f/L)		
Day 1	84.12 ± 7.95	84.44 ± 7.45
Day 30	84.68 ± 7.40	84.20 ± 7.59
Day 60	85.40 ± 6.92	84.44 ± 8.09
MCH (pg)		
Day 1	27.04 ± 3.19	27.20 ± 3.00
Day 30	27.52 ± 3.04	27.44 ± 3.15
Day 60	27.56 ± 2.79	27.363 ± 3.12
MCHC (g/L)		
Day 1	321.12 ± 12.85	322.40 ± 11.84
Day 30	324.28 ± 12.91	325.24 ± 13.32
Day 60	323.12 ± 12.87	321.72 ± 13.81
RDW (%)		
Day 1	13.87 ± 1.93	13.62 ± 1.47
Day 30	14.02 ± 2.01	13.65 ± 1.40
Day 60	13.83 ± 1.89	13.60 ± 1.50
White cell count (×10^9^ L)		
Day 1	7.18 ± 1.77	7.16 ± 1.45
Day 30	6.97 ± 1.58	7.85 ± 2.15
Day 60	6.89 ± 1.80	6.85 ± 1.28
Neutrophils (×10^9^ L)	3.88 ± 1.68	3.80 ± 1.24
Day 1	3.84 ± 1.29	4.67 ± 2.01
Day 30	3.79 ± 1.32	3.70 ± 1.31
Day 60		
Lymphocytes (×10^9^ L)		
Day 1	2.53 ± 0.53	2.58 ± 0.71
Day 30	2.40 ± 0.56	2.44 ± 0.61
Day 60	2.38 ± 0.63	2.35 ± 0.52
Monocytes (×10^9^ L)		
Day 1	0.51 ± 0.18	0.52 ± 0.13
Day 30	0.49 ± 0.19	0.50 ± 0.17
Day 60	0.52 ± 0.20	0.48 ± 0.11
Eosinophils (×10^9^ L)		
Day 1	0.24 ± 0.14	0.24 ± 0.17
Day 30	0.20 ± 0.12	0.21 ± 0.13
Day 60	0.19 ± 0.10	0.21 ± 0.11
Basophils (×10^9^ L)		
Day 1	0.09 ± 0.03	0.08 ± 0.04
Day 30	0.07 ± 0.05	0.09 ± 0.04
Day 60	0.08 ± 0.04	0.09 ± 0.03
Platelets (×10^9^ L)		
Day 1	307.80 ± 69.97	310.68 ± 78.80
Day 30	296.28 ± 71.44	296.28 ± 71.44
Day 60	302.88 ± 65.15	302.88 ± 65.15
ESR (mm/h)		
Day 1	10.28 ± 7.19	10.72 ± 6.23
Day 30	12.96 ± 12.17	15.92 ± 18.69
Day 60	12.40 ± 10.07	10.64 ± 9.95

**Table 8:** Plasma lipid profiles (mean ± SD).

	OPP treatment (n = 25)	Control treatment (n = 25)
Total cholesterol, TC (mmol/L)		
Day 1	4.99 ± 0.71	4.83 ± 0.74
Day 30	5.17 ± 0.75	4.97 ± 0.72
Day 60	4.94 ± 0.70^1^	5.16 ± 0.82
Triacylglycerol, TAG (mmol/L)		
Day 1	1.09 ± 0.69	1.15 ± 0.61
Day 30	1.14 ± 0.61	1.06 ± 0.54
Day 60	0.94 ± 0.40	0.97 ± 0.47
HDL-C (mmol/L)		
Day 1^2^	1.36 ± 0.32	1.29 ± 0.30
Day 30	1.36 ± 0.30	1.31 ± 0.29
Day 60	1.26 ± 0.26	1.32 ± 0.29
LDL-C (mmol/L)		
Day 1	3.13 ± 0.66	3.01 ± 0.66
Day 30	3.29 ± 0.77	3.17 ± 0.70
Day 60	3.25 ± 0.73^3^	3.40 ± 0.79
Total cholesterol/HDL ratio		
Day 1	3.89 ± 1.13	3.93 ± 1.11
Day 30	4.03 ± 1.22	4.00 ± 1.18
Day 60	4.10 ± 1.21	4.12 ± 1.24

^1^Although time × treatment interaction was significant, p = 0.001 (2-factor repeated measures ANOVA) and TC was significantly lower for OPP compared to control treatment (at day 60), based on Wilcoxon signed rank test (p = 0.025), no significant difference of total cholesterol level was found between OPP and control treatments at day 1, 30 and 60 following Bonferroni correction.

^2^Baseline HDL-C level was significantly different between treatments, p = 0.038 (Wilcoxon signed rank test). The value was further used as covariate in repeated measures. Although the time × treatment analysis was significant, p = 0.011 (2-factor repeated measures ANOVA), no significant difference of HDL-C level was found between treatments at day 30 and 60 based on Wilcoxon signed rank test. Similarly, no significant difference of HDL-C level was found between treatments at day 30 and 60 after Bonferroni correction.

^3^Although the time × treatment interaction was significant, p = 0.018 (2-factor repeated measures ANOVA) and LDL-C was significantly lower for OPP compared to control treatment (at day 60) based on Wilcoxon signed rank test (p = 0.04), no significant difference of LDL-C level was found between OPP and control treatments at day 1, 30 and 60 following Bonferroni correction.

**Table 9:** Endocrinology profiles (mean ± SD).

	OPP treatment (n = 25)	Control treatment (n = 25)
Serum insulin (µU/mL)		
Day 1	9.56 ± 6.38	9.52 ± 6.95
Day 30	9.24 ± 5.43	9.16 ± 5.47
Day 60	9.76 ± 5.97	9.60 ± 4.90
Plasma glucose (mmol/L)		
Day 1	4.80 ± 0.29	4.75 ± 0.26
Day 30	4.66 ± 0.35	4.66 ± 0.27
Day 60	4.63 ± 0.30	4.56 ± 0.33

**Table 10:** Special chemistry profile (mean ± SD).

	OPP treatment (n = 25)	Control treatment (n = 25)
Serum hsCRP (mg/L)		
Day 1	1.68 ± 2.41	1.58 ± 2.42
Day 30	2.53 ± 3.97	1.50 ± 2.34
Day 60	3.71 ± 6.12	2.66 ± 4.70
Whole blood HbA1c (%)		
Day 1	5.41 ± 0.25	5.43 ± 0.26
Day 30	5.43 ± 0.24	5.40 ± 0.24
Day 60	5.48 ± 0.24	5.47 ± 0.25
Urine albumin (mg/L)		
Day 1	19.52 ± 44.54	13.91 ± 25.76
Day 30	19.56 ± 9.67	12.48 ± 8.00
Day 60	16.51 ± 31.66	14.37 ± 10.10
ACR (Alb/mmol Cr)		
Day 1	3.13 ± 0.66	3.01 ± 0.66
Day 30	3.29 ± 0.77	3.17 ± 0.70
Day 60	3.25 ± 0.73^3^	3.40 ± 0.79

**Table 11:** Electrolyte profiles (mean ± SD).

	OPP treatment (n = 25)	Control treatment (n = 25)
Plasma sodium (mmol/L)		
Day 1	139.92 ± 1.71	140.04 ± 1.49
Day 30	140.12 ± 1.94	139.56 ± 2.08
Day 60	138.48 ± 1.56	138.48 ± 1.08
Plasma potassium (mmol/L)		
Day 1	4.30 ± 0.37	4.24 ± 0.31
Day 30	4.36 ± 0.42	4.54 ± 2.00
Day 60	4.20 ± 0.36	4.05 ± 0.37
Plasma chloride (mmol/L)		
Day 1	104.08 ± 1.68	104.52 ± 2.22
Day 30	102.36 ± 1.89	102.76 ± 1.74
Day 60	102.64 ± 1.73	102.40 ± 1.50

**Figure 1:**
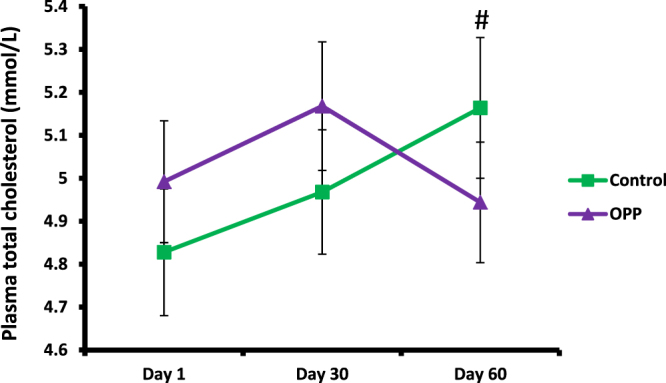
Total cholesterol level in plasma following OPP and control treatments for 60 days. ^#^Time × treatment interaction was significant, p = 0.001 (2-factor repeated measures ANOVA). Based on Wilcoxon signed rank test, TC was significantly lower for OPP treatment compared to control treatment (at day 60), p = 0.025. However, following Bonferroni correction, no significant difference of total cholesterol level was found between OPP and control treatments at day 1, 30 and 60.

**Figure 2:**
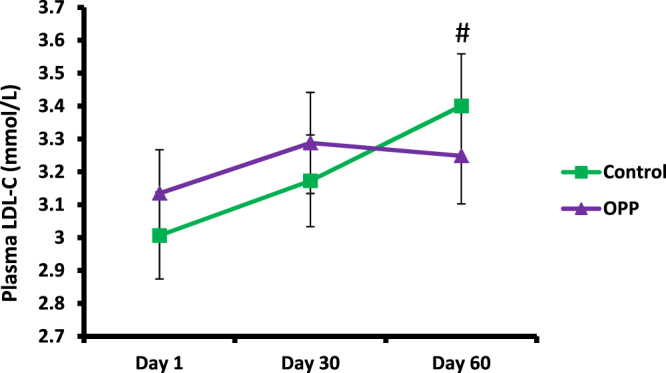
LDL-C level following OPP and control treatments for 60 days. ^#^Time × treatment interaction was significant, p = 0.018 (2-factor repeated measures ANOVA). Based on Wilcoxon signed rank test, LDL-C was significantly lower after OPP compared to control treatment (at day 60), p = 0.04. However, following Bonferroni correction, no significant difference of LDL-C level was found between OPP and control treatments at day 1, 30 and 60.

**Figure 3:**
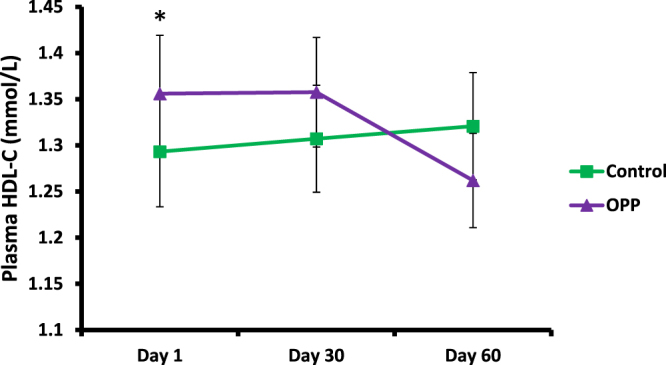
HDL-C level following OPP and control treatments for 60 days. *Baseline HDL-C level was significantly different between treatments, p = 0.038 (Wilcoxon’s signed-rank test). Value was further used as covariates in repeated measures. Although the time × treatment analysis was significant, p = 0.011 (2-factor repeated measures ANOVA), no significant difference of HDL-C level was found between treatments at day 30 and 60 (Wilcoxon signed rank test). Similarly, no significant difference of HDL-C level was found between treatments at day 30 and 60 after Bonferroni correction.

The OPP dose used in the trial resulted in lower plasma TC (Fig. 1 and Table 8). After 60 days of OPP supplementation, plasma TC level in the OPP treatment group was significantly lower compared to the control treatment (p = 0.025) based on the Wilcoxon signed rank test. Plasma LDL-C was also significantly lower when volunteers were supplemented with OPP (p = 0.04), (Fig. 2 and Table 8). As several dependent and independent statistical tests were performed in this study, in order to rule out false positive results and increase the stringency of the test, Bonferroni correction was applied. Following the Bonferroni correction, the decrease in TC and LDL-C was deemed to be not significant. There was no significant difference of HDL-C (Fig. 3), TAG and TC/HDL ratio (Table 8) between OPP and control treatments based on the Wilcoxon signed rank test and the Bonferroni correction.

## Discussion

Dosage of the OPP was determined based on the safety and functionality of a similar intake of GAE from pomegranate juice. Previous investigation demonstrated that pomegranate dietary supplement is safe when ingested by healthy human volunteers in amounts up to 1420 mg/day, providing a total of 870 mg GAE/day^21^. No adverse effects or changes in haematology, serum chemistry, or urinalyses related to the pomegranate dietary supplement consumption were observed during the trial period. In the current trial, volunteers were given a total of 300 mL of OPP with a concentration of 1500 mg/L GAE (150 mL of OPP in the morning session and another 150 mL of OPP in the afternoon session). This provided a total of 450 mg GAE/day to each volunteer for 60 days.

The dose was selected based on translation of the effective dose used in our previous pre-clinical trials on animal models supplemented with OPP where positive effects on clinical biochemistry profiles were demonstrated^2,11,12,14,16^. Most clinical trials have attempted to deliver large quantities of individual polyphenols to overcome the issues of rapid polyphenol clearance in the human circulation^24^. Supplementation of phenolics in high doses appears to be the only reliable method to achieve measurable concentrations approaching those seen in *in vitro* and *in vivo* animal studies, thus enabling investigators to observe any beneficial effects that may occur due to the supplementation.

In most clinical trials investigating the physiological effects of phenolic substances, safety aspects are rarely documented. It is further suggested that detailed safety profiles should be included when findings are reported and published^24^. Following OPP supplementation in our trial, no AE/SAE or changes in haematology, serum and plasma chemistry, as well as urinalyses were observed. Moreover, none of the volunteers showed any allergic reactions towards OPP supplementation. All clinical biochemistry liver profiles were in the normal range according to the standard clinical safety references, indicating no toxic effects from OPP supplementation. In our previous investigations on animals, no toxicity effects from OPP supplementations (up to 2400 mg GAE/L dose) were found, as far as the histology, haematology and clinical biochemistry profiles were concerned^2,11,12,14,16^. Therefore, it can be concluded that OPP at the dose tested in the current trial is generally safe for human consumption.

Introduction of the control treatment arm into the trial was necessary as previously recommended^25,26^. Furthermore, a much longer wash out period (four-weeks) was implemented in the current trial, to ensure the duration was sufficient to avoid any possible effects of the repeated ingestions^25^. Although it has been reported that a one-week wash out period is adequate^27,28^ given that most polyphenols are rapidly metabolized and reach maximum concentration within hours^20^, this does not take into account the fact that phenolic extracts including pomegranate supplement and OPP contain other compounds such as fibre, sugars, organic acids and vitamins besides phenolics. A longer washout period is thus advantageous.

Most of the human trials investigating the effects of phenolics and polyphenols were focused on evaluating beneficial effects. Safety issues such as side effects, acute or chronic/long-term effects have rarely been reported in the literature^24^. A primary aim of this trial however, was to ascertain the safety of consuming OPP.

Among all results, we carefully evaluated the response of plasma lipid profiles (TC, LDL-C and HDL-C), glucose and insulin levels towards OPP supplementation, given that OPP had demonstrated positive effects in lowering lipid and diabetic profiles in our previous studies using animal models^11^. The OPP dose utilized in the current trial reduced plasma TC and LDL-C compared to the control treatment. Although the reduction was significant based on the Wilcoxon signed rank test, the application of the Bonferroni correction suggested that the results were not significant. Previous pre-clinical studies demonstrated the ability of OPP in lowering plasma TC^11,16^ in diabetic rats. Additionally, previous studies showed that genes involved in cholesterol biosynthesis in BALB/c mice were down-regulated by OPP, hence eliciting a hypocholesterolaemic effect^12,14^. Further clinical tests to specifically confirm the effect of OPP on lipid profiles in humans with raised TC may be necessary.

Blood lipid profiles (TAG, TC, LDL-C, HDL-C) have been routinely used as indicators in evaluating the physiological potential of phenolic supplementations in human trials. In most cases, no improvement has been clearly demonstrated in healthy subjects when phenolic substances were supplemented^29^. A previous human intervention trial also found no significant differences in lipid profiles namely TAG, TC, LDL-C, and HDL-C between treatment and control groups following red wine consumption for two weeks^30^. However, the phenolic content used in that trial was much lower (62 mg epicatechin equivalent/day) compared to our trial. Even larger doses such as 852 mg GAE/day of total phenolic intake of cranberry juice for the same period did not significantly alter plasma lipid profiles in healthy population^31^.

Our findings are in agreement with a previous trial where 49 males and 11 females were supplemented with 544 mg/day polyphenol extract powder (dissolved as drinks) for two months^32^. There were no significant differences in serum TAG, TC, HDL-C and LDL-C after consumption of the polyphenols in two different intervention groups. In another human trial, supplementation of 710 mg/day or 1420 mg/day of pomegranate polyphenol extract in the form of capsules for four weeks in 64 healthy subjects also did not significantly improve plasma TAG, TC, HDL-C and LDL-C levels^21^. Supplementation with 120 mg/day of isoflavones through consumption of soy preparation powder for 12 weeks significantly reduced TC, LDL-C and cholesterol/HDL ratio but not HDL-C levels. These effects were however demonstrated in diabetic volunteers^27^. In a later study which applied the same recruitment period and the same type of isoflavone preparation but at a slightly higher dose (132 mg/day), TAG, TC, LDL-C and HDL-C levels were not significantly changed as well^33^. Theoretically as an antioxidant, OPP may provide positive effects in improving the lipid profiles in volunteers with raised TC levels. However, in the current trial, no significant differences in TAG and HDL-C levels between OPP and control treatments were observed. It is important to note that the volunteers were healthy and their lipid profiles were already in the normal range and hence it is not surprising that no significant changes were observed following OPP supplementation.

The effect of phenolic supplementations on lipid profiles in humans is rather unique. Several contradictory findings from the literature suggest that recruitment periods, doses and forms of phenolic supplementations, as well as health condition of recruited volunteers affect the results of the supplementations. A short supplementation period of 150 mg/day of polyphenols rich pine bark extract in capsule form for six weeks significantly improved LDL-C and HDL-C profiles in 25 healthy subjects^34^. In comparison to the placebo treatment, supplementation with the same type of polyphenols^34^ but at slightly lower dose for a much longer period (125 mg/day) in diabetics with mild hypertension resulted in significantly lower LDL-C levels after 8 and 12 weeks^35^. A significant LDL-C lowering effect was also demonstrated in diabetic volunteers even after four weeks of supplementation with 321 mg/day of flavanol-rich drinks^36^. However, a two-week supplementation period of 800 mg/day polyphenol capsules did not significantly alter blood lipid profiles in 35 healthy male subjects^28^. Even high dose of phenolic substance at longer period did not improve lipid profiles, as previously demonstrated in obese subjects who received 800 mg/day of catechin supplement capsules for eight weeks^37^.

In the current study, following 60 days of OPP supplementation, plasma glucose levels declined gradually from their respective baseline values. However, this observation did not reflect any of the glucose-reducing effects as previously suggested in our animal studies^11,16^, since a similar trend was even demonstrated in the controls. Nevertheless, no significant changes in glucose and insulin levels were observed after 60 days of OPP supplementation. Furthermore, there was no significant difference in plasma glucose at day 30 or 60 between both OPP and control treatments. However, this is not surprising as this trial was with healthy volunteers and reduction in glucose levels was not anticipated. OPP may have elicited a different effect in diabetic or pre-diabetic volunteers.

Most of the glucose lowering effects by polyphenols in healthy populations were demonstrated in the postprandial trial, where subjects were challenged with the oral glucose tolerance test (OGTT) coupled with polyphenol treatments^38,39^. No significant effects were however demonstrated during long-term supplementation trials in healthy populations^19,21,30,37,40^ and even in pathological populations^18,27,33,36,40,41^. Similar to other antioxidants, the effects of polyphenol treatments are likely to be influenced by doses, durations and delivery forms of supplementations, as well as the health status of the studied populations. Higher polyphenol treatments such as ≈1500 mg/day for two weeks significantly reduced plasma glucose level in type 2 diabetic volunteers^42^. However, the actual effects of the polyphenol treatment in the study were not clearly established since the diabetic volunteers were also prescribed with the oral blood glucose-lowering drugs, although the comparison was made with the control treatment^42^. Furthermore, the relationship between blood glucose and insulin levels in diabetic volunteers has not yet been conclusive since some polyphenol treatments resulted in significantly improved insulin profiles but not glucose levels^19^.

Although there are no clear guidelines in determining the upper-tolerable intake of phenolic supplementations, future human trials should establish the effective minimum dose of phenolic intake that could significantly improve desired clinical profiles. Our phase one clinical trial demonstrated the safety and physiological effects of OPP, as supported by the current available data of clinical biochemistry profiles. Supplementation of 450 mg GAE/day OPP is found to be safe for human consumption. Although the results were not significant following Bonferroni correction, the observed decrease in TC and LDL-C suggests that at appropriate doses, OPP may provide several beneficial effects in improving lipid profiles in humans and the current observations justify the need to continue with dose-exploration trials of OPP in future.

## Electronic supplementary material

Clinical Trial Protocol
