# Supplementary material for: A phase I single-blind clinical trial to evaluate the safety of oil palm phenolics (OPP) supplementation in healthy volunteers
Source: Sci Rep. 2018 May 29;8:8217. doi: 10.1038/s41598-018-26384-7 (PMC5974131; doi:10.1038/s41598-018-26384-7)
Supplement: Supplementary file 1 — Clinical Trial Protocol [file 41598_2018_26384_MOESM1_ESM.docx]

**SUPPLEMENTARY INFORMATION**

**CLINICAL TRIAL PROTOCOL**

**A phase I single-blind clinical trial to evaluate the safety of oil palm phenolics (OPP) supplementation in healthy volunteers**

**Authors:**

Syed Fairus^1*^, Soon-Sen Leow^1^, Naina Mohamed Isa^2^, Yew-Ai Tan^1^, Kalyana Sundram^3^ & Ravigadevi Sambanthamurthi^1^.

**Affiliation:**

^1^Malaysian Palm Oil Board (MPOB), No. 6 Persiaran Institusi, Bandar Baru Bangi, 43000 Kajang, Selangor, Malaysia.

^2^Universiti Kebangsaan Malaysia Medical Centre (UKMMC), Jalan Yaacob Latif, Bandar Tun Razak, 56000, Cheras, Kuala Lumpur, Malaysia.

^3^Malaysian Palm Oil Council (MPOC), 2^nd^ Floor, Wisma Sawit, Lot 6, SS6, Jalan Perbandaran, 47301, Kelana Jaya, Selangor, Malaysia.

***Corresponding author:**

Syed Fairus, Tel: +603-8769 4604, Fax: +603-8926 5926, email: [syfairus@mpob.gov.my](mailto:syfairus@mpob.gov.my)

**Co-authors email:** [ssleow@mpob.gov.my](mailto:ssleow@mpob.gov.my) (Soon-Sen Leow), [isanaina@yahoo.co.uk](mailto:isanaina@yahoo.co.uk) (Naina Mohamed Isa), [yewaitan@gmail.com](mailto:yewaitan@gmail.com) (Yew-Ai Tan), [kalyana@mpoc.org.my](mailto:kalyana@mpoc.org.my) (Kalyana Sundram), [ravigadevi@gmail.com](mailto:ravigadevi@gmail.com) (Ravigadevi Sambanthamurthi).

**Project summary**

Our previous pre-clinical trial demonstrated that OPP treatment is safe and provided several beneficial physiological effects. Therefore, a phase one clinical trial was conducted to evaluate the safety and effects of OPP in healthy volunteers. In this single-blind design trial, after an overnight fast, 25 healthy volunteers (11 males, 14 females) were supplemented with 150 mL of 450 mg gallic acid equivalent (GAE)/day of OPP or 150 mL of control drinks (drinking water), twice per day for a 60-day period. The dose was selected based on translation of the effective dose used in our previous pre-clinical trials on animal models supplemented with OPP where positive effects on clinical biochemistry profiles were demonstrated. All volunteers consumed both drinks in front of the investigator. The trial design allowed the investigators to detect early physiological outcomes from OPP intake. Fasting blood (20 mL) and urine samples were collected at days 1, 30 and 60 where medical examination was performed during these interventions. A four-week (28 days) wash-out period was allowed between both treatments. All 25 volunteers completed the recruitment where every clinical biochemistry profiles observed throughout the control and OPP treatments period were in the normal range with no major adverse effect (AE) or serious adverse effect (SAE) was observed. Additionally, OPP supplementation resulted in improvement of total cholesterol and LDL-C levels, compared to the control treatment. Nevertheless, there was no significant difference of HDL-C, TAG and TC/HDL ratio between OPP and control treatments. The outcomes support our previous observations that OPP is safe and may have a protective role in reducing cholesterol levels.

**General information**

1. **Protocol title** : Supplementation of palm fruit juice in normal and pre-diabetic

human volunteers (submitted to Medical Research Ethics

Committee, Malaysian Ministry of Health).

1. **Protocol code** : OPP 100(H)-2009 (Malaysian Ministry of Health), and

Trial Reference No: ACTRN 12611001122943; Australian New- Zealand Clinical Trial Registry (ANZCTR)

1. **Ethical approval** : Medical Research Ethics Committee, Malaysian Ministry of

Health, NMRR-08-1618-3108 (approval date: 5^th^ July 2011). The official approval letter is attached herewith for reference (Appendix section).

1. **Sponsors :**
2. Malaysian Palm Oil Board (MPOB). No. 6, Persiaran Institusi, Bandar Baru Bangi, 43000, Kajang, Selangor, Malaysia.
3. ScienceFund, Malaysian Ministry of Agriculture and Agro-Based Industry (0104031002). Blok 4G1 Wisma Tani, No.28 Persiaran Perdana, Presint 4, Pusat Pentadbiran Kerajaan Persekutuan, 62624 Putrajaya Malaysia
4. **Investigators details, address and roles:**
5. Dr Syed Fairus bin Syed Abu Bakar

Senior Research Officer,

Malaysian Palm Oil Board (MPOB),

No. 6 Persiaran Institusi, Bandar Baru Bangi, 43000 Kajang, Selangor, Malaysia.

Tel: +603-8769 4604

Role: Principal Investigator

1. Dr Leow Soon Sen

Senior Research Officer,

Malaysian Palm Oil Board (MPOB),

No. 6 Persiaran Institusi, Bandar Baru Bangi, 43000 Kajang, Selangor, Malaysia.

Tel: +603-8769 4552

Role: Investigator

1. Assoc. Prof Dr Isa Naina Mohamed,

Universiti Kebangsaan Malaysia Medical Centre (UKMMC), Jalan Yaacob Latif, Bandar Tun Razak, 56000, Cheras, Kuala Lumpur, Malaysia.

Tel: +603-9289 7281

Role: Investigator and Medical Officer

1. Dr Tan Yew Ai

Senior Principle Research Officer,

Malaysian Palm Oil Board (MPOB),

No. 6 Persiaran Institusi, Bandar Baru Bangi, 43000 Kajang, Selangor, Malaysia.

Tel: +603-8769 4604

Role: Investigator

1. Dr Kalyana Sundram

Chief Executive Officer,

Malaysian Palm Oil Council (MPOC), 2nd Floor, Wisma Sawit, Lot 6, SS6, Jalan Perbandaran, 47301, Kelana Jaya, Selangor, Malaysia

Tel: +603-7803 4248

Role: Investigator

1. Dr Ravigadevi Sambanthamurthi

Senior Principle Research Officer,

Malaysian Palm Oil Board (MPOB),

No. 6 Persiaran Institusi, Bandar Baru Bangi, 43000 Kajang, Selangor, Malaysia.

Tel: +603-8769 4498

Role: Investigator

1. **Clinical laboratories**:
2. Metabolic Laboratory

Advanced Biotechnology and Breeding Unit (ABBC)

Malaysian Palm Oil Board (MPOB)

6, Persiaran Institusi

Bandar Baru Bangi

43000 Kajang

Selangor, Malaysia.

Tel: 03-8769 4548

Fax: 03-8926 1995

1. Gribbles Pathology

13-1-1, 1st Floor

Jln Medan PB 1A

Pusat Bandar Bangi, Section 9

43650 Bandar Baru Bangi

Selangor, Malaysia.

Tel: 03-8925 6172

Fax: 03-8925 2759

1. **Rationale and background information**

Previous pre-clinical investigations discovered that supplementation of OPP manages to improve circulating blood glucose and plasma TG in Nile rats with early diabetes (mean glucose >110 mg/dL) [1-2]. Additionally, a long-term intake of OPP protected healthy, young Nile rats against diabetes onset, as measured by glucose, blood lipids, and weight of livers and kidneys. These observations are probably due to the antioxidant protection by phenolics compound in the OPP liquid and their protection of ß-cells in the pancreatic islet against oxidative stress. Therefore, the ß-cells will be able to maintain its integrity and consistently produce insulin. In order to produce continuous and sufficient insulin for the body, the ß-cell must be protected against oxidation stress.

Given that the OPP contains several types of phenolics compounds, we hypothesize that supplementation of the OPP may provide an antioxidant protection against oxidative stress in diabetic subject. Additionally, our previous observations in animal model showed that genes involved in cholesterol biosynthesis in BALB/c mice were down-regulated by OPP, hence eliciting a hypocholesterolaemic effect [3-4]. Other positives physiological effects were also demonstrated in much earlier [5] and following pre-clinical studies by the Malaysian Palm Oil Board (MPOB).

Currently, there is still no clinical data from human study on the physiological effects of OPP. Moreover, the number of human study investigating the antioxidant potential of several common phenolics compounds such as quercetin and catechin is still limited. One of the goals in delaying the onset of type 2 DM is to preserve the function of ß-cells in the pancreatic islet. Based on the current evidence from the preliminary studies on OPP, we hypothesize that supplementation of OPP to humans at pre-diabetic state may prevent or delay the development of type 2 DM.

However, in order to understand the anti-diabetic effect of OPP in humans, we need to firstly establish our knowledge on the physiological effects of this compound to normal human subjects. Under physiological condition, OPP may improve the antioxidant status profiles of healthy subjects. In pre-diabetic subjects, improvement of antioxidant status may have a positive influence on plasma diabetes profiles, since many scientific evidences demonstrate that there is an inverse relationship between antioxidant status and incidence of DM (11-12). Therefore, we proposed a human nutrition study to evaluate the antioxidant effects of OPP (which contained high concentration of palm phenolics compound) on healthy human subjects. Data from this study would hopefully assists us in understanding the therapeutical roles of palm phenolics on humans under normal and pathological conditions.

This study will involve the recruitment of healthy normal human volunteers. The study will be started with 25 healthy volunteers where they will be supplemented with OPP or control drinks for a period of 60 days. Blood samples will be analysed for several antioxidant status, lipid and diabetic profiles at baseline and after supplementation (on day-30 and 60).

**References:**

1. Sambanthamurthi, R. et al. Positive outcomes of oil palm phenolics on degenerative diseases in animal models. Br. J. Nutr. 106,1664-1675 (2011).
2. Bolsinger, J., Pronzcuk, A., Sambanthamurthi, R., Hayes, K.C. Anti diabetic effect of palm fruit juice in the nile rat (Arvicanthis niloticus). J. Nutr. Sci. 3(e5), 1-11 (2014).
3. Leow, S.S., Sekaran, S.D., Sundram, K., Tan, Y.A. & Sambanthamurthi, R. Differential transcriptomic profiles effected by oil palm phenolics indicate novel health outcome. BMC Genomics. 12, 432 (2011).
4. Leow, S.S., Sekaran, S.D., Sundram, K., Tan, Y.A. & Sambanthamurthi, R. Oil palm phenolics attenuate changes caused by an atherogenic diet in mice. Eur J Nutr. 52, 443-446 (2013).
5. Balasundram, N., Tan, Y.A., Sambanthamurthi, R., Sundram, K. & Samman, S. Antioxidant properties of palm fruit extracts. Asia Pac. J. Clin. Nutr. 4,319-324 (2005).
6. Leow, S.S., Sekaran, S.D., Sundram, K., Tan, Y.A. & Sambanthamurthi, R. Differential transcriptomic profiles effected by oil palm phenolics indicate novel health outcome. BMC Genomics. 12, 432 (2011).
7. Leow, S.S., Sekaran, S.D., Sundram, K., Tan, Y.A. & Sambanthamurthi, R. Oil palm phenolics attenuate changes caused by an atherogenic diet in mice. Eur J Nutr. 52, 443-446 (2013).
8. Idris, C.A.C. et al. Oil palm phenolics and vitamin E reduce atherosclerosis in rabbits. J Func Foods. 7,541-550 (2014).

1. Patten, G.S., Abeywardena, M.Y., Sundram, K., Tan, Y.A., Sambanthamurthi, R. Effect of oil palm phenolics on gastrointestinal transit, contractility and motility in the rat. J Func Foods. 17, 928-937 (2016).
2. Leow, S.S., Bolsinger, J., Pronczuk, A., Hayes, K.C. & Sambanthamurthi, R. Hepatic transcriptome implications for palm fruit juice deterrence of type 2 diabetes mellitus in young male Nile rats. Genes & Nutrition. 11, 29 (2016).
3. Coskun, O., Kanter, M., Korkmaz, A. and Oter, S. Quercetin, a flavonoid antioxidant, prevents and protects streptozotocin-induced oxidative stress and ß-cell damage in rat pancreas. Pharmacol. Res. 51:117-123 (2005).
4. Rosenblat, M., Hayek, T. and Aviram, M. Anti-oxidative effects of pomegranate juice (PJ) consumption by diabetic patients on serum and on macrophages. Atherosclerosis. 187:363-371 (2006).

**8. Study objectives**

1. To evaluate the physiological effects of OPP supplementation in normal healthy human volunteers.
2. To investigate and evaluate any Adverse Effect/Event (AE), Adverse Reaction (AR) or Serious Adverse Event (SAE) or Serious Adverse Reaction (SAR) following OPP supplementation (up-to 60 days supplementation).

**9. Study protocol**

**9.1 Study design**

The trial is registered with the Australian New Zealand Clinical Trial Registry (ANZCTR) database (Trial Reference No: ACTRN 12611001122943). Trial progress and related information can be accessed via www.anzctr.org.au/. This was a single-blind, selected, placebo-controlled designed to evaluate the antioxidant potential of OPP supplementation on healthy human volunteers, with comparison to placebo treatment, for a period of 60 days.

25 healthy volunteers were enrolled into the study. Volunteers were requested to fast overnight, for at least 10 hours. Volunteers attended clinic for baseline measurement of plasma clinical profiles (day 1). Fasting blood (20 mL) and urine samples were taken. Urine samples were also collected before bleeding session. Volunteers were assigned into two (2) intervention groups, where one group (treatment group) was given 150 mL of OPP (containing 1500 mg/L GAE OPP), twice per day. The other group (placebo group), was given 150 mL of control drinks, twice per day. The trial design allowed the Investigators to detect early physiological outcomes from OPP intake (treatment group). Additionally, application of the current design in the study minimized the study error or bias.

During the 60-day intervention period, volunteers were allowed to maintain their habitual diets. Throughout the whole intervention, fasting blood (20 mL) and urine samples were again taken at day 30 and 60. In every clinic visit, the measurement of blood pressure (systolic/diastolic) and anthropometry (body weight and height) were recorded. For each volunteer, four (4) weeks (28 days) of wash-out period was allowed between study interventions. The schematic diagram of the study design for Phase 1 is shown in **FIGURE 1.**


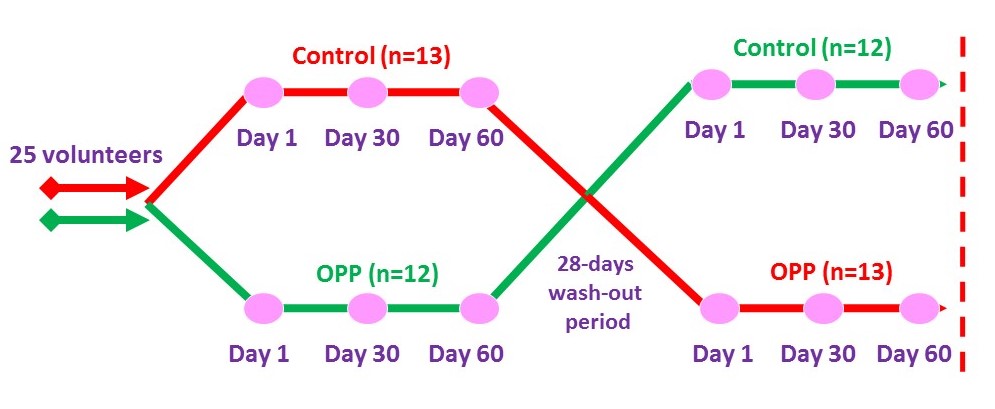


**Figure 1**

**9.1.2 Recruitment of volunteers**

25 healthy volunteers, consisting of 18 males and 17 females were recruited from MPOB, to participate in the Phase 1 Study. Volunteers were selected according to several inclusion and exclusion criteria, as follows:

**9.1.2.1 Inclusion criteria**

1. Written informed consent obtained from volunteers.
2. Volunteers age between 20 and 40 y.
3. Normal levels of fasting plasma TG (<1.9 mmol/L) and cholesterol (3.9 to 5.72 mmol/L).
4. Normal level of fasting plasma glucose (<6.1 mmol/L or 109.8 mg/dL, according to the WHO 1999 Report (63).
5. Having a good record of compliance.

**9.1.2.2 Exclusion criteria**

1. Smoking
2. Habitual alcohol consumption
3. Consumption of any supplement vitamins especially antioxidant rich supplement vitamin.
4. Taking any types of drugs/pharmacological agent or traditional herbs preparation.
5. Pregnant.
6. Lactating.
7. Taking contraceptives during the time of study enrollment.
8. Having any cardiovascular diseases (CVDs), cancer or any type of pathological conditions.

**9.1.2.3 Rights of volunteers to drop out of the study**

Volunteers were free to drop out from the study at anytime for any reason. Volunteers may also be dropped out from the study at any time at the discretion of the investigator. If there is any, the reason for the drop out must be obtained and stated in the report.

**9.2 Methodology**

**9.2.1 Blood and urine sampling**

Blood sample will be drawn from the volunteers by an experienced and well-trained phlebotomist using a syringe. A 20 mL blood will be then transferred into blood collection tube with ethylenediamine tetra acetic acid (EDTA) and without EDTA. The EDTA treated blood sample will be centrifuged at 3000 x g for 20 minutes at 7⁰C to obtain plasma sample. Urine sample will also be taken prior to the blood sampling.

**9.2.2 Laboratory analyses**

| **Clinical profiles** | **Tests** | **Clinical profiles** | **Tests** |
| --- | --- | --- | --- |
| ***1. Lipid profiles*** | Total cholesterol (TC)  Triacylglycerol (TAG)  LDL-C  HDL-C  TC/HDL ratio | ***2. Endocrinology*** | Serum insulin  Plasma glucose |
|  |  | ***4. Electrolytes*** | Plasma sodium  Plasma potassium |
| ***3.Special chemistry*** | Serum hsCRP  Whole blood HbA1c  Urine albumin  Urine creatinine |  |  |
|  |  | ***6. Liver function*** | Plasma total protein  Plasma albumin  Plasma globulin  Plasma ALP  Plasma total bilirubin  Plasma GGT  Plasma AST  Plasma ALT |
| ***5. Renal function*** | Plasma urea  Plasma creatinine  Plasma uric acid  Plasma calcium  Plasma phosphate |  |  |
|  |  | ***8.Blood pressure*** | Systolic  Diastolic  Pulse rate |
| ***7. Haematology*** | RBC  PCV  MCV  MCH  MCHC  RDW  White cell count  Neutrophils  Lymphocytes  Monocytes  Eosinophil  Basophils  Platelets  ESR |  |  |

**9.2.3 Statistical method**

Wilcoxon-Signed Test was performed to compare significance of differences between parameters of interest before (baseline, day 1) and after treatment (days 30, and 60) for each treatment. Effects of treatment on parameters of interest were analyzed for their time x treatment interaction, using Two-factor repeated measures analysis of variance (ANOVA) with an interaction term to detect whether there was a significant difference of plasma and urine profiles between OPP and control treatments..If there was any significant time x treatment interaction, the value at the specific day of treatment was extensively compared using Wilcoxon-Signed Rank Test. To increase the stringency of the analysis, Bonferroni correction for multiple testing was applied. Statistical analysis was performed using Statistical Package for Social Sciences (SPSS®) for WINDOWS software (Version 10.0, SPSS Inc. Chicago, USA) and MS Excel 2003 (Microsoft Corp. California, USA). The MS Excel software was used for tabulation of statistical charts. The SPSS® software was utilized for calculation of plasma profiles and analyses of Repeated Measures ANOVA, Bonferroni and Wilcoxon-Signed Rank Test. Values were considered significant at P<0.05.
